# Recombinant Rat CC10 Protein Inhibits PDGF-Induced Airway Smooth Muscle Cells Proliferation and Migration

**DOI:** 10.1155/2013/690937

**Published:** 2013-09-11

**Authors:** Ying Wei, Yu-Dong Xu, Lei-Miao Yin, Yu Wang, Jun Ran, Qi Liu, Zi-Feng Ma, Yan-Yan Liu, Yong-Qing Yang

**Affiliations:** Laboratory of Molecular Biology, Yueyang Hospital of Integrated Traditional Chinese and Western Medicine, Shanghai University of Traditional Chinese Medicine, 650 South Wanping Road, Shanghai 200030, China

## Abstract

Abnormal migration and proliferation of airway smooth muscle cells (ASMCs) in the airway cause airway wall thickening, which is strongly related with the development of airway remodeling in asthma. Clara cell 10 kDa protein (CC10), which is secreted by the epithelial clara cells of the pulmonary airways, plays an important role in the regulation of immunological and inflammatory processes. Previous studies suggested that CC10 protein had great protective effects against inflammation in asthma. However, the effects of CC10 protein on ASMCs migration and proliferation in airway remodeling were poorly understood. In this study, we constructed the pET-22b-CC10 recombinant plasmid, induced expression and purified the recombinant rat CC10 protein from *E. coli* by Ni^2+^ affinity chromatography and ion exchange chromatography purification. We investigated the effect of recombinant rat CC10 protein on platelet-derived growth factor (PDGF)-BB-induced ASMCs proliferation and migration. Our results demonstrated that the recombinant CC10 protein could inhibit PDGF-BB-induced cell viability, proliferation and migration. Western blot analysis showed that PDGF-BB-induced activation of cyclin D1 was inhibited by CC10. These findings implicated that CC10 could inhibit increased ASMCs proliferation, and migration induced by PDGF-BB, and this suppression effect might be associated with inhibition of cyclin D1 expression, which might offer hope for the future treatment of airway remodeling.

## 1. Introduction

Asthma is a chronic airway inflammatory disease which has the characteristics of airway hyperresponsiveness, airway inflammation, and airway remodeling. Persistent inflammation in airway may lead to structural changes known as airway remodeling [1]. More and more evidence indicated that airway remodeling is closely related to the progression of airway hyperresponsiveness and the severity of asthma. One of the dominant structural changes of airway remodeling is the increase in airway smooth muscle (ASM) mass [2]. It was demonstrated that a histological thickness of smooth muscle was increased in asthmatic airways [3]. Increased ASMCs proliferation and migration is responsible for this ASM thickness change and contributes to the remodeling of the smooth muscle within the airway wall [4]. Increased proliferation and migration decreased pulmonary function in asthmatic patients [4–6]. Considering that airway remodeling in asthma is poorly responsive to current therapies [2], it will be valuable to search for new molecules to prevent airway remodeling.

It appeared that PDGF had a prominent role in promoting smooth muscle proliferation and migration. The PDGF family is composed of five different dimeric isoforms: PDGF-AA, PDGF-AB, PDGF-BB, PDGF-CC, and PDGF-DD [7]. PDGF, which was secreted by epithelial cells and inflammatory cells from asthmatic airways [8–10], had been shown to be elevated in the lungs of asthmatics and was thought to contribute to airway remodeling and ASM proliferation [11, 12].

Clara cell 10 kDa protein (CC10), which is produced by the nonciliated, nonmucous, secretory epithelial clara cells of the pulmonary airways, was first identified in lung lavage by Singh and colleagues [13–15]. CC10 consists of a homodimer of 70–77 amino acid polypeptides held together by two disulfide bridges arranged in antiparallel fashion [16]. Previous studies has suggested that CC10 have great protective effects against inflammation in asthma [17–21]. However, the effects of CC10 protein on airway remodeling were poorly understood. In this study, we constructed the pET-22b-CC10 recombinant plasmid, induced expression, and purified the recombinant rat CC10 protein from *E. coli* by Ni^2+^ affinity chromatography and ion exchange chromatography purification. We investigated the effect of recombinant rat CC10 protein on PDGF-BB-induced ASMCs proliferation and migration. We showed here that recombinant rat CC10 protein had inhibitory effect on PDGF-BB-induced ASMCs proliferation and migration in airway remodeling.

## 2. Materials and Methods

### 2.1. Reagents

*E. coli* strain BL21 (DE3) was a generous gift from Shanghai National Engineering Center for Biochips. pET-22b plasmid was a gift from the Pharmaceutical Institute of Chinese Academy of Sciences. WST-1 Cell Proliferation and Cytotoxicity Assay Kit and the fluorescent dye DAPI were purchased from Beyotime. Ni^2+^ Sepharose 6 Fast Flow and Q Sepharose Fast Flow were purchased from GE Healthcare. PDGF-BB was purchased from R&D Systems. Dulbecco's modified Eagle's medium (DMEM), PBS, and penicillin streptomycin solution were purchased from Hyclone. Fetal bovine serum (FBS) and 0.25% Trypsin-EDTA solution were purchased from Gibco. 96-well plates, 6-well plates, and Boyden chamber were purchased from Corning Costar. E-Plate 16 was purchased from Roche. Antibody against cyclin D1 was purchased from Epitomic. Antibody against *α*-smooth muscle actin and TRITC-conjugated goat anti-rabbit IgG were purchased from Abcam.

### 2.2. Recombinant Plasmid Construction

Rat CC10 coding region sequences were obtained from Gene Bank, NM_013051.1 and were amplified by PCR. The 5′ primer used was 5′-GGAATTCCATATGAAGATCGCCATCACAATCA-3′ and the 3′ primer used was 5′-CCGCTCGAGGACTCTTAAATCTTGCTCACAC-3′. The recombinant pET-22b-CC10 plasmids containing the synthetic CC10 gene were cloned through Nde I and Xho I restriction sites and were transformed into host cell BL21 (DE3). The positive colonies containing the recombinant pET-22b-CC10 plasmids were screened by colony PCR and sequencing analysis. 

### 2.3. Expression and Purification of the Recombinant Rat CC10 Protein

For recombinant protein expression, positive *E. coli* strain containing recombinant pET-22b-CC10 plasmid was induced by addition of isopropyl-*β*-D-thiogalactopyranoside (IPTG). After IPTG induction, the bacterial pellets were collected by centrifugation. The bacterial pellets were resuspended in buffer A (20 mM sodium phosphate, 500 mM NaCl, 20 mM imidazole, pH 8.0) and lysed by lysozyme. The lysate was centrifuged, and the supernatant was collected. The recombinant rat CC10 protein in the supernatant was purified by affinity chromatography purification using Ni^2+^ Sepharose 6 Fast Flow beads and ion exchange chromatography purification using Q Sepharose Fast Flow beads by gravity flow according to the manufacturer's instructions. SDS-PAGE gels and Western blotting were used for purity analysis and antigenic activity assay.

### 2.4. Isolation, Characterization, and Culture of Rat ASMCs

ASMCs were isolated from the airway of SD rat conforming to the regulations of the State Science and Technology Commission. The rat tracheal was dissected under sterile conditions in normal saline solution. The smooth muscle layer was dissected free of the adherent connective tissue and cartilage, and the epithelial layer was removed. The smooth muscle section was then chopped finely into 1 mm × 1 mm tissues and incubated in 12 cm^2^ flasks for 3 h in 3 mL DMEM supplemented with 10% FBS, 100 U/mL penicillin, and 0.1 mg/mL streptomycin at 37°C in humidified air containing 5% CO_2_. Then, the smooth muscle tissues were cultured in 5 mL 10% FBS-DMEM for 5 days and changed with fresh culture medium every three days. ASMCs were grown until they formed a confluence and passaged with 0.25% trypsin-EDTA solution. The characteristic of ASMCs were determined by immunofluorescence with antibodies against *α*-smooth muscle actin. All experiments were performed with ASMCs from passages 6 to 12.

### 2.5. Cell Proliferation/Viability Assay

To estimate cell proliferation/viability, we performed WST-1 assay and Roche Real-Time Cell Analyzer (RTCA) DP assay. WST-1 cell proliferation assay which was designed to measure the relative proliferation rates of cells in culture is commonly used for the nonradioactive quantification of cellular proliferation. WST-1 reagent is a colorimetric assay, and it is an alternative product for MTT and XTT. The principle of WST-1 assay is that it could be converted into a colored dye by mitochondrial dehydrogenase enzymes which are proportional to the cell number. Compared with the traditional label-based endpoint assays like WST-1, the Roche RTCA assay has emerged as an alternative noninvasive and label-free approach to assess cellular proliferation, migration, and invasion in real-time on a cell culture level with specific plates designed for different research purposes [22, 23]. The RTCA assay measures impedance changes in a meshwork of interdigitated gold microelectrodes located at the well bottom of E-plate 16. These changes are caused by the gradual increase of electrode surface occupation by proliferated cells during the course of time, and thus, can provide an index of cell viability and proliferation [24].

ASMCs were seeded into 96 well plate or E-Plate 16 at a density of 3000 cells/well with DMEM containing 10% FBS. The cells were growth arrested by withdrawing serum for 24 h. Then, cells were treated with increasing doses of CC10 protein (1 ng/mL, 10 ng/mL, 50 ng/mL, 100 ng/mL, 200 ng/mL, 400 ng/mL, and 800 ng/mL) 1 h before stimulation with PDGF-BB (25 ng/mL) in 1% FBS-DMEM for 24 h. To compare the effect of CC10 protein on PDGF-BB-induced ASMCs proliferation/viability with hydrocortisone (HC, a widely used drug in treating asthma), we performed WST-1 assay and the Roche RTCA DP assay. ASMCs were treated with CC10 protein (400 ng/mL) or HC (1 mg/mL) 1 h before stimulation with PDGF-BB (25 ng/mL) in 1% FBS-DMEM for 24 h, 48 h, and 72 h. The cell proliferation/viability was determined using the Roche RTCA DP assay/WST-1 assay according to the manufacturer's instructions. Data were generated from ASMCs with 4 replicates. 

### 2.6. Boyden Chamber Migration Assay

The migration assay was performed using the Transwell system. The lower compartment was filled with 0.6 mL of DMEM containing 1% FBS with 25 ng/mL PDGF-BB alone or together with CC10 (400 ng/mL) or HC (1 mg/mL). ASMCs (1 × 10^5^) were resuspended in 0.1 mL of DMEM and placed in the upper part of the Transwell plate. Cells were incubated for 8 h in a humidified atmosphere of 5% CO_2_ at 37°C. ASMCs were fixed with 4% paraformaldehyde and stained with trypan blue for 10 min. ASMCs on the upper surface of the filter were mechanically removed by wiping with a cotton swab, and the migrated cells were determined by counting the cells that migrated to the lower side of the filter using a microscope. Six randomly selected fields were counted, and each sample was assayed in triplicate.

### 2.7. Western Blot Analysis

ASMCs were seeded into 6-well plates with 10% FBS-DMEM. The cells were growth arrested by withdrawing serum for 24 h. Then, cells were treated with CC10 protein (400 ng/mL) or HC (1 mg/mL) 1 h before stimulation with PDGF-BB (25 ng/mL) in 1% FBS-DMEM for 24 h and 48 h. The total protein was extracted using RIPA protein extraction reagent. Immunoblotting was performed with cyclin D1 antibody (1 : 10000 diluted), *β*-actin antibody (1 : 10000 diluted), and secondary peroxidase-conjugated IgG (1 : 10000 diluted). The immunoreactivity was detected by chemiluminescence as previously described [25]. The protein bands were quantified using a Bio-Rad Image Lab calibrated densitometer.

### 2.8. Statistical Analysis

Results are expressed as mean ± SD values. Statistical analyses of the data were performed by one-way analysis of variance (ANOVA) for multiple comparisons followed by the LSD test for comparisons between groups. Values of *P* < 0.05 were considered statistically significant. 

## 3. Results 

### 3.1. Expression and Purification of Recombinant CC10 Protein

**Figure 1:**
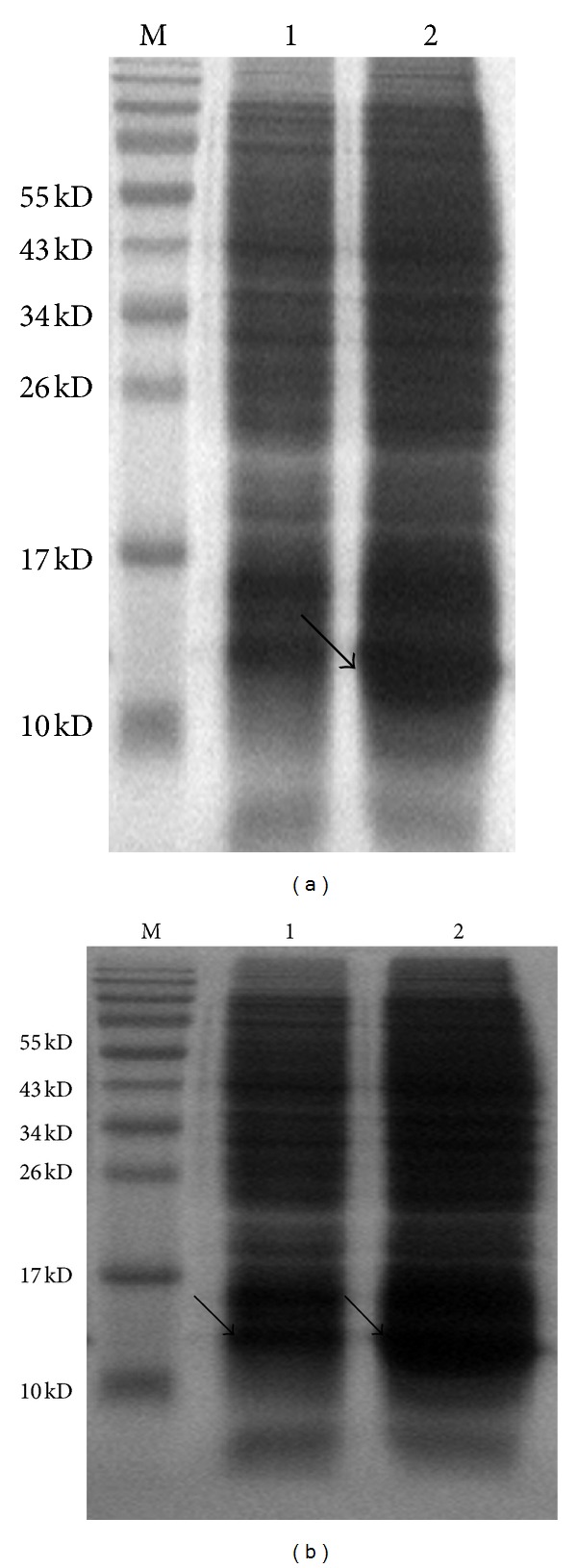
Induced expression and soluble analysis of recombinant rat CC10 protein. (a) Induced expression of recombinant CC10 protein; 1: uninduced recombinant pET-22b-CC10 culture; 2: induced recombinant pET-22b-CC10 culture. (b) Soluble analysis of the induced expression of recombinant CC10 protein; 1: insoluble pellet of the induced recombinant pET-22b-CC10 culture; 2: clarified supernatant of the induced recombinant pET-22b-CC10 culture.

**Figure 2:**
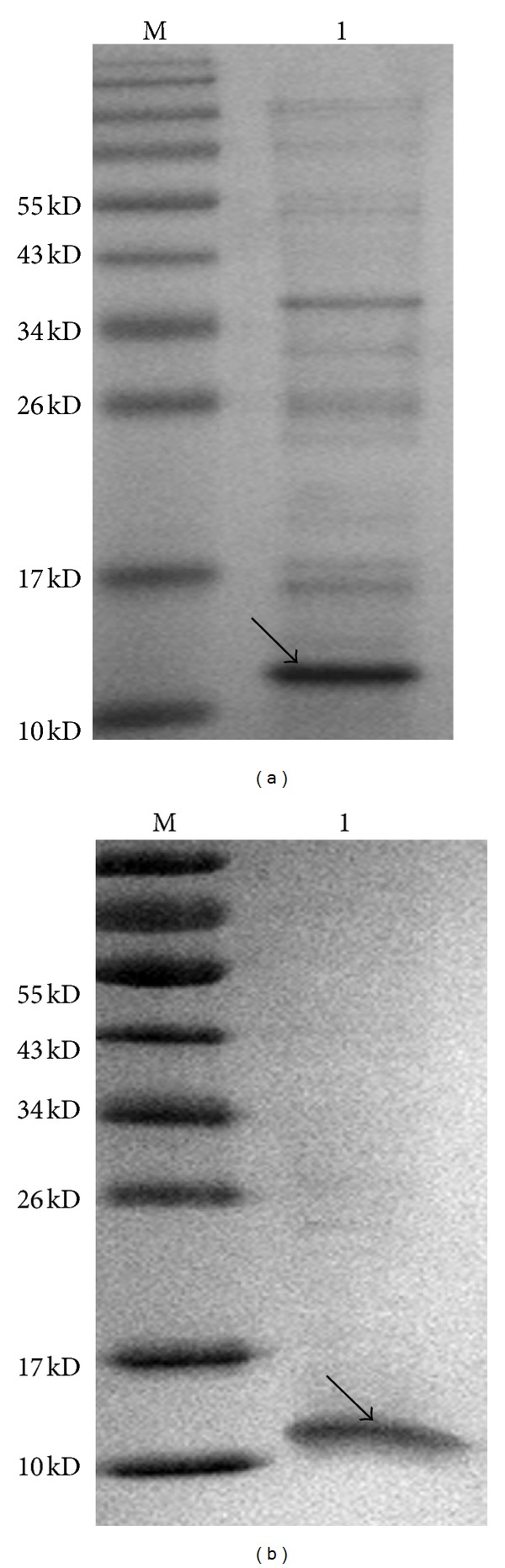
Analysis of the recombinant rat CC10 protein purity by SDS-PAGE; (a) purity of recombinant CC10 protein after affinity chromatography purification; (b) purity of recombinant CC10 protein after affinity chromatography and ion exchange chromatography purification.

The expression of CC10 was induced by IPTG, and the optimal condition at which we arrived was induction of CC10 for 4 h with 0.4 mM IPTG at 21°C (Figure 1(a)). SDS-PAGE analysis of the supernatant and pellet showed that the recombinant CC10 protein was mainly expressed in soluble form (Figure 1(b)). The recombinant rat CC10 protein was purified by affinity chromatography purification using Ni^2+^ Sepharose 6 Fast Flow beads and ion exchange chromatography purification using Q Sepharose Fast Flow beads. After this two-step purification, we obtained the recombinant rat CC10 protein in high purity with a single visible band (Figure 2). Western blot analysis with antibodies against CC10 demonstrated its antigenic activity.

### 3.2. Recombinant Rat CC10 Protein Inhibits PDGF-BB-Induced ASMCs Viability/Proliferation

**Figure 3:**
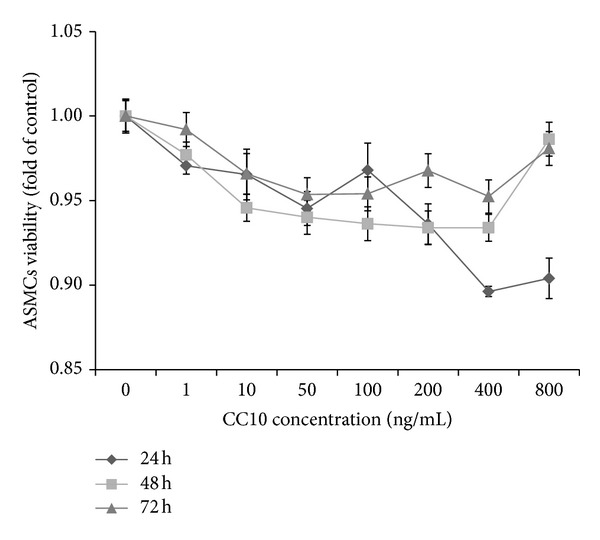
Dose dependent inhibitory effect of CC10 protein on PDGF-BB-induced ASMCs viability. Data was generated from ASMCs with 4 replicates. Percentage of viability was obtained by comparing OD values to that of ASMCs treated in parallel with PDGF-BB.

We examined the effect of increasing doses of CC10 (1 ng/mL, 10 ng/mL, 50 ng/mL, 100 ng/mL, 200 ng/mL, 400 ng/mL, and 800 ng/mL) on PDGF-BB-induced ASMCs viability. In the WST-1 assay system, ASMCs showed a 134% increase in cellular viability compared to the PBS control after stimulation with 25 ng/mL PDGF-BB for 24 h (*P* < 0.01). CC10 protein inhibited ASMCs viability between doses of 1 ng/mL and 800 ng/mL (Figure 3). 400 ng/mL CC10 protein had the strongest inhibitory effect on ASMCs viability among the doses, and we chose this dose for the follow-up study.

**Figure 4:**
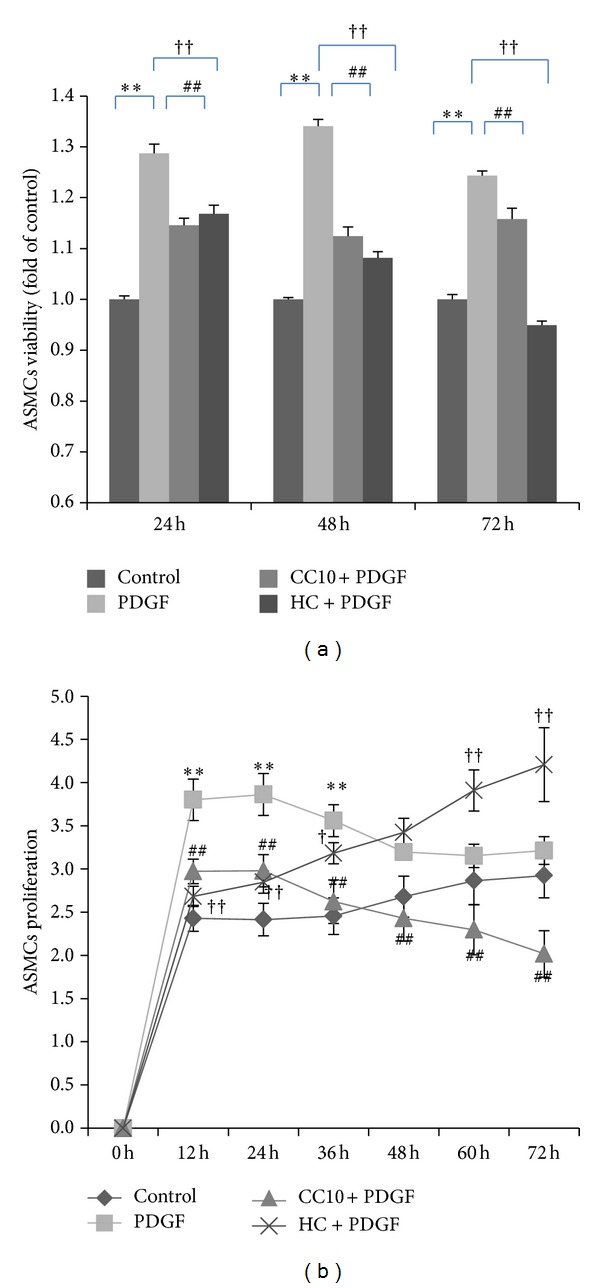
Effects of recombinant rat CC10 protein on PDGF-BB-induced ASMCs viability/proliferation. (a) Comparison of the effect of CC10 protein on ASMCs viability to that of HC by WST-1 assay. (b) Comparison of the effect of CC10 protein on ASMCs proliferation with that of HC by Roche RTCA DP assay. Data was generated from ASMCs with 4 replicates. Percentage of viability was obtained by comparing OD values to that of ASMCs in a control group treated in parallel with PBS. ASMCs proliferation variation was obtained by calculating the changes of ASMCs to baseline. Data was shown as means ± SD values. **P < 0.01 PDGF group versus control, *P < 0.05 PDGF group versus control, ^##^ *P* < 0.01 CC10 group versus PDGF group, ^††^ *P* < 0.01 HC group versus PDGF group, and ^†^ *P* < 0.05 HC group versus PDGF group.

We compared the effect of 400 ng/mL CC10 on PDGF-BB-induced ASMCs viability and proliferation with HC. In the WST-1 assay system, ASMCs showed 129%, 134%, and 124% increases in cellular viability after stimulation with 25 ng/mL PDGF-BB for 24 h, 48 h, and 72 h compared to the PBS control (*P* < 0.01). Pretreatment with 400 ng/mL CC10 suppressed PDGF-BB-induced cellular viability to about 89%, 84%, and 93% for 24 h, 48 h, and 72 h compared to the PDGF stimulation (*P* < 0.01). Pretreatment with 1 mg/mL HC suppressed PDGF-BB-induced cellular viability to about 90%, 81%, and 76% for 24 h, 48 h, and 72 h compared to the PDGF group (*P* < 0.01) (Figure 4(a)). In the WST-1 assay system, the effect of CC10 protein was the same as that of HC at 24 h (*P* > 0.05).

In the Roche RTCA DP assay system, we observed the real-time effect of CC10 protein on PDGF-BB-induced ASMCs proliferation for 72 h. We chose data of 12 h, 24 h, 36 h, 48 h, 60 h, and 72 h for analysis. Stimulation with PDGF-BB (25 ng/mL) increased ASMCs proliferation to 157% (*P* < 0.01) for 12 h, 160% (*P* < 0.01) for 24 h, 145% (*P* < 0.01) for 36 h, 119% (*P* < 0.05) for 48 h, and 110% for 60 h and 72 h compared to the PBS control. Pretreatment with CC10 (400 ng/mL) suppressed PDGF-BB-induced cell proliferation to 78% for 12 h, 77% for 24 h, 74% for 36 h, 76% for 48 h, 73% for 60 h, and 63% for 72 h compared to the PDGF group (*P* < 0.01). Pretreatment with 1 mg/mL HC suppressed PDGF-BB-induced cellular proliferation to about 71% for 12 h (*P* < 0.01), 74% for 24 h (*P* < 0.01), and 89% for 36 h (*P* < 0.05) compared to the PDGF group (Figure 4(b)). In this experimental system, we found out that both CC10 protein and HC had an inhibitory effect on PDGF-BB-induced ASMCs proliferation. In the first 36 h after PDGF-BB induction, CC10 protein and HC had the same inhibitory effect on cell proliferation (*P* > 0.05). In the time after 48 h, HC manifested promotion of ASMCs proliferation, and this promotion of cell proliferation could reach 131% compared to control (*P* < 0.01). These results demonstrated that CC10 (400 ng/mL) negatively regulates cellular proliferation in response to PDGF signaling, and this inhibitory effect was stronger than HC after 36 h.

### 3.3. Recombinant Rat CC10 Protein Suppresses PDGF-BB-Induced ASMCs Migration

**Figure 5:**
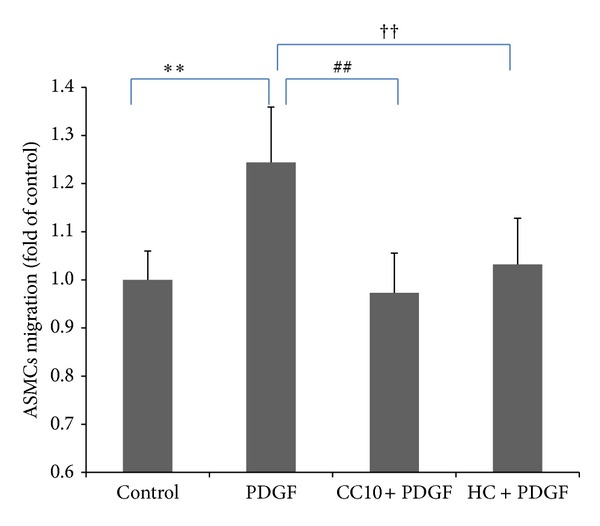
The effects of recombinant rat CC10 protein on PDGF-BB-induced ASMCs migration. The migration assay was performed using the Transwell system. Migrated cells were determined by counting the cells that migrated to the lower side of the filter using a microscope. Six randomly selected fields were counted, and each sample was assayed in triplicate. Data was shown as means ± SD values. **P < 0.01 PDGF group versus control, ^##^ *P* < 0.01 CC10 group versus PDGF group, and ^††^ *P* < 0.01 HC group versus PDGF group.

In the Boyden chamber assay system, stimulation with PDGF-BB (25 ng/mL) for 8 h increased ASMCs migration to about 124% compared with the PBS control (*P* < 0.01). This migration response to PDGF-BB stimulation was significantly suppressed by CC10 (400 ng/mL) to 78% compared to the PDGF group (*P* < 0.01). HC (1 mg/mL) could inhibit this PDGF-induced cell migration to about 83% compared to the PDGF group (*P* < 0.01). It was worth noting that this inhibitory effect of CC10 protein on PDGF-induced ASMCs migration was stronger than that of HC (Figure 5).

### 3.4. Recombinant Rat CC10 Protein Suppresses PDGF-BB-Induced Cyclin D1 Expression

**Figure 6:**
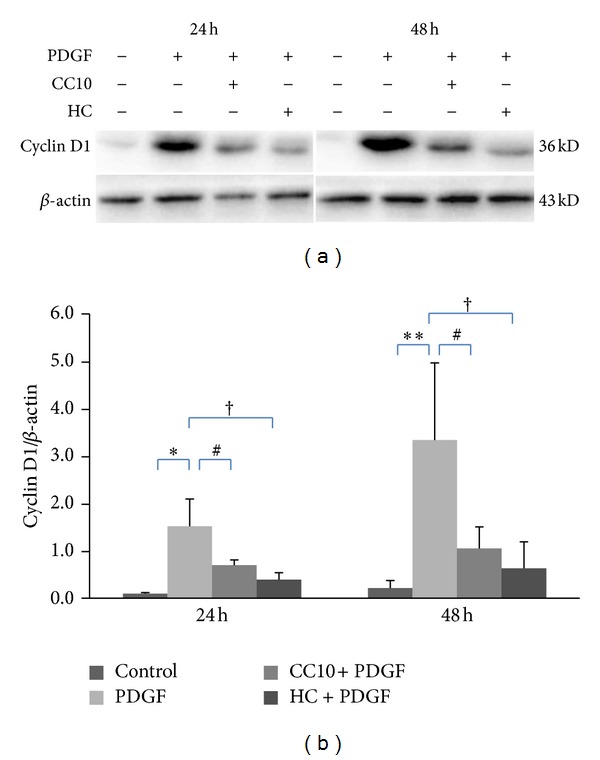
The effects of recombinant rat CC10 protein on PDGF-BB-induced cyclin D1 expression. (a) Western blot analysis of cyclin D1 expression. (b) The expression of cyclin D1 was quantified and represented as band intensity normalized to *β*-actin. Data was shown as means ± SD values. **P < 0.01 PDGF group versus control, *P < 0.05 PDGF group versus control, ^#^ *P* < 0.01 CC10 group versus PDGF group, and ^†^ *P* < 0.05 HC group versus PDGF group.

To further elucidate the inhibitory effect of CC10 protein on ASMCs proliferation and migration, we investigated the effect of CC10 protein on cell cycle regulatory protein cyclin D1 expression. Cyclin D1 expression was increased significantly in ASMCs after stimulation with PDGF-BB (25 ng/mL) for 24 h and 48 h compared to the PBS control (*P* < 0.05). Pretreatment with CC10 (400 ng/mL) reduced PDGF-BB-induced expression of cyclin D1 (Figure 6).

## 4. Discussion

CC10 is a steroid-inducible, homodimeric, and low molecular weight secretory protein. It is suggested that natural CC10 is difficult to obtain in amounts sufficient for the detailed characterization of its biological properties. It has been recognized that homodimer proteins with two interchain disulfide bonds are very difficult to express in their natural quaternary structure in bacterial hosts [26]. The pET system is the most powerful system developed for the cloning and expression of recombinant proteins in *E. coli.* In our experimental system, CC10 DNA was cloned in pET-22b plasmid and transferred into BL21 (DE3). Recombinant rat CC10 protein expression was induced by IPTG. Our results demonstrated that after IPTG induction, recombinant CC10 had been stably expressed in the pET-22b recombinant plasmid. However, in our early experiments, more than half of this protein was expressed in the insoluble pellet. Lower growth temperatures have been shown to prevent aggregation into insoluble inclusion bodies [27]. To improve the yield of active soluble recombinant CC10 protein, we performed temperature and time course experiments which aimed to optimize the induction temperature and time as well as the IPTG concentration. After a series of trials, we arrived at the optimal conditions induction of the recombinant vector with 0.4 mM IPTG for 4 h at 21°C. For recombinant rat CC10 protein purification, we used affinity chromatography and ion-exchange chromatography purification in order to obtain high purity recombinant protein. We chose Ni^2+^ Sepharose 6 Fast Flow and Q Sepharose Fast Flow beads according to the characteristics of the pET-22b plasmid and the CC10 protein as well as the elution buffer used in affinity chromatography purification. After the two-step purification, we obtained recombinant rat CC10 protein with high purity.

ASM proliferation and migration is a major pathological component in airway remodeling of asthma. In vitro studies demonstrated that ASMCS from asthmatics exhibited increased proliferation capacity compared to myocytes from nonasthmatic individuals [28, 29]. ASMCs in asthmatic airways remodeled inward towards the mucosal layer, and this pathological change was thought to result from ASMCs migration in response to growth and chemokinetic factors [30]. PDGF-BB was one of those factors responsible for the ASMCs proliferation and migration in airway remodeling [31]. In our experiments, we chose PDGF-BB as an inducer to induce ASMCs proliferation and migration. Stimulation with PDGF-BB significantly promoted ASMCs proliferation both in the WST-1 system and the Roche RTCA DP assay system. Previous studies demonstrated that ASMCs could be changed from a quiescent to a migratory and proliferative phenotype by exposure to a variety of growth factors and cytokines in asthma [32–34]. In our Boyden chamber assay system, stimulation with PDGF-BB significantly improved the ASMCs migration. Our results demonstrated that PDGF-BB significantly induced ASMCs proliferation and migration, which was one of the key pathological changes of airway remodeling in asthma, and our discovery was the same as results of former studies [30, 31]. Frequent stimulation of ASMCS by growth factors, contractile agonists, and inflammatory mediators could cause airway remodeling and airflow obstruction, some of which can become irreversible [34]. Decreasing cell proliferation and reducing cell migration into the smooth muscle layer are two mechanisms which could limit the accumulation of smooth muscle in airway remodeling [35]. In our study, we observed the effect of the CC10 protein on ASMCs proliferation and migration. Stimulation with PDGF-BB significantly promoted ASMCs proliferation and migration, and CC10 protein significantly inhibited this proliferation and migration, indicating that CC10 might be used to prevent ASMCs proliferation and migration in airway remodeling in asthma. The cell cycle regulatory protein cyclin D1 has been the most widely studied cyclin in ASM biology [34]. Our study showed here that cyclin D1 expression was activated in ASMCs by PDGF-BB stimulation, which was in accordance with other studies [36, 37]. The activation of cyclin D1 is necessary for transition through the restriction point into DNA synthesis [38] and is associated with ASMCs proliferation [37, 39]. In the present study, PDGF-BB induced a high level of cyclin D1 activation, and CC10 protein inhibited PDGF-BB-induced cyclin D1 expression significantly in ASMCs. These results revealed that the antiproliferative and antimigrative effect of CC10 protein might be associated with downregulation of cyclin D1 expression.

HC is one of the commonly used glucocorticoids in asthma treatment, and long term usage of it might bring some side effects, especially, for children with asthma. CC10 is a naturally secreted protein in our body with almost the same inhibitory effect as that of HC on PDGF-BB-induced ASMCs proliferation and migration, and to some extent, it might be developed into a substitute for HC in inhibiting disorders regarding abnormal ASMCs proliferation and migration.

 In our experiment, we investigated the effect of recombinant rat CC10 protein on PDGF-BB-induced ASMCs proliferation and migration. Our results showed that CC10 protein could inhibit ASMCs proliferation and migration, which were responsible for airway wall thickness in airway remodeling. The suppression of ASMCs proliferation and migration might be associated with downregulation of cyclin D1 expression. Our discovery might offer hope for the future treatment of airway remodeling.
